# Corrigendum: Unveiling Interindividual Variability of Human Fibroblast Innate Immune Response Using Robust Cell-Based Protocols

**DOI:** 10.3389/fimmu.2021.685768

**Published:** 2021-04-26

**Authors:** Audrey Chansard, Nelly Dubrulle, Mathilde Poujol de Molliens, Pierre B. Falanga, Tharshana Stephen, Milena Hasan, Ger van Zandbergen, Nathalie Aulner, Spencer L. Shorte, Brigitte David-Watine, the Milieu Intérieur Consortium, Laurent Abel

**Affiliations:** ^1^ UTechS Photonic BioImaging, C2RT, Institut Pasteur, Paris, France; ^2^ UTechS Cytometry and Biomarkers, CRT, Institut Pasteur, Paris, France; ^3^ Division of Immunology, Paul-Ehrlich-Institut, Federal Institute for Vaccines and Biomedicines, Langen, Germany; ^4^ Pasteur Joint International Research Unit Ai3D, Institut Pasteur Korea, Seongnam-si, South Korea; ^5^ Unité INSERM U 1223, Institut Pasteur, Paris, France; ^6^ Unité Biologie et Génétique de la Paroi Bactérienne, Institut Pasteur, Paris, France; ^7^ CNRS UMR2001, Paris, France; ^8^ INSERM, Équipe Avenir, Paris, France

**Keywords:** immortalization, HSV-1, FACS, cytokines, human primary cells, NF-kB, TLR (Toll like receptors), innate immunity

“The Milieu Intérieur Consortium” was not included in the list of the authors in the published article as it should be.

Further, in the original article, there was an error. An outdated version of the Milieu Intérieur Consortium appears at the end of the article.

A correction has been made to the *Consortium* section:

“*The Milieu Intérieur* Consortium is composed of the team leaders: Laurent Abel (Hôpital Necker), Andres Alcover, Hugues Aschard, Kalla Astrom (Lund University), Philippe Bousso, Pierre Bruhns, Ana Cumano, Caroline Demangel, Ludovic Deriano, James Di Santo, Françoise Dromer, Gérard Eberl, Jost Enninga, Jacques Fellay (EPFL, Lausanne), Ivo Gomperts-Boneca, Milena Hasan, Serge Hercberg (Université Paris 13), Olivier Lantz (Institut Curie), Hugo Mouquet, Etienne Patin, Sandra Pellegrini, Stanislas Pol (Hôpital Côchin), Antonio Rausell (INSERM UMR 1163 – Institut Imagine), Lars Rogge, Anavaj Sakuntabhai, Olivier Schwartz, Benno Schwikowski, Spencer Shorte, Frédéric Tangy, Antoine Toubert (Hôpital Saint-Louis), Mathilde Trouvier (Université Paris 13), Marie-Noëlle Ungeheuer, Darragh Duffy§, Matthew L. Albert (In Sitro)§, Lluis Quintana-Murci§”.

¶ unless otherwise indicated, partners are located at Institut Pasteur, Paris

§ co-coordinators of the Milieu Intérieur Consortium.

Additional information can be found at http://www.milieuinterieur.fr/

The authors apologize for these errors and state that these do not change the scientific conclusions of the article in any way. The original article has been updated.

