# Supplementary figures and images for: Unveiling Interindividual Variability of Human Fibroblast Innate Immune Response Using Robust Cell-Based Protocols
Source: Front Immunol. 2021 Jan 11;11:569331. doi: 10.3389/fimmu.2020.569331 (PMC7829859; doi:10.3389/fimmu.2020.569331)

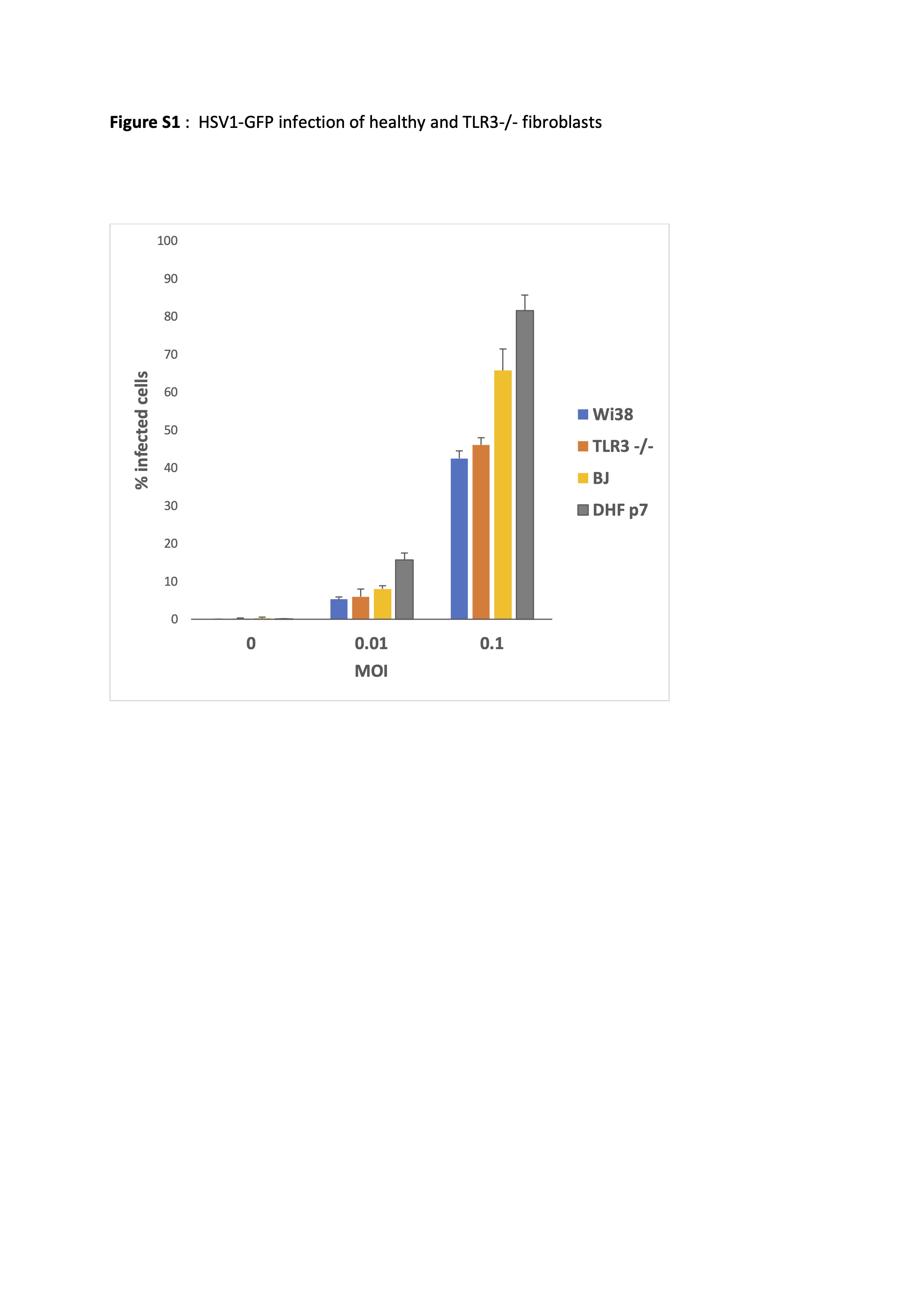

Supplement: Supplementary file 1 [file Image_1.jpeg]

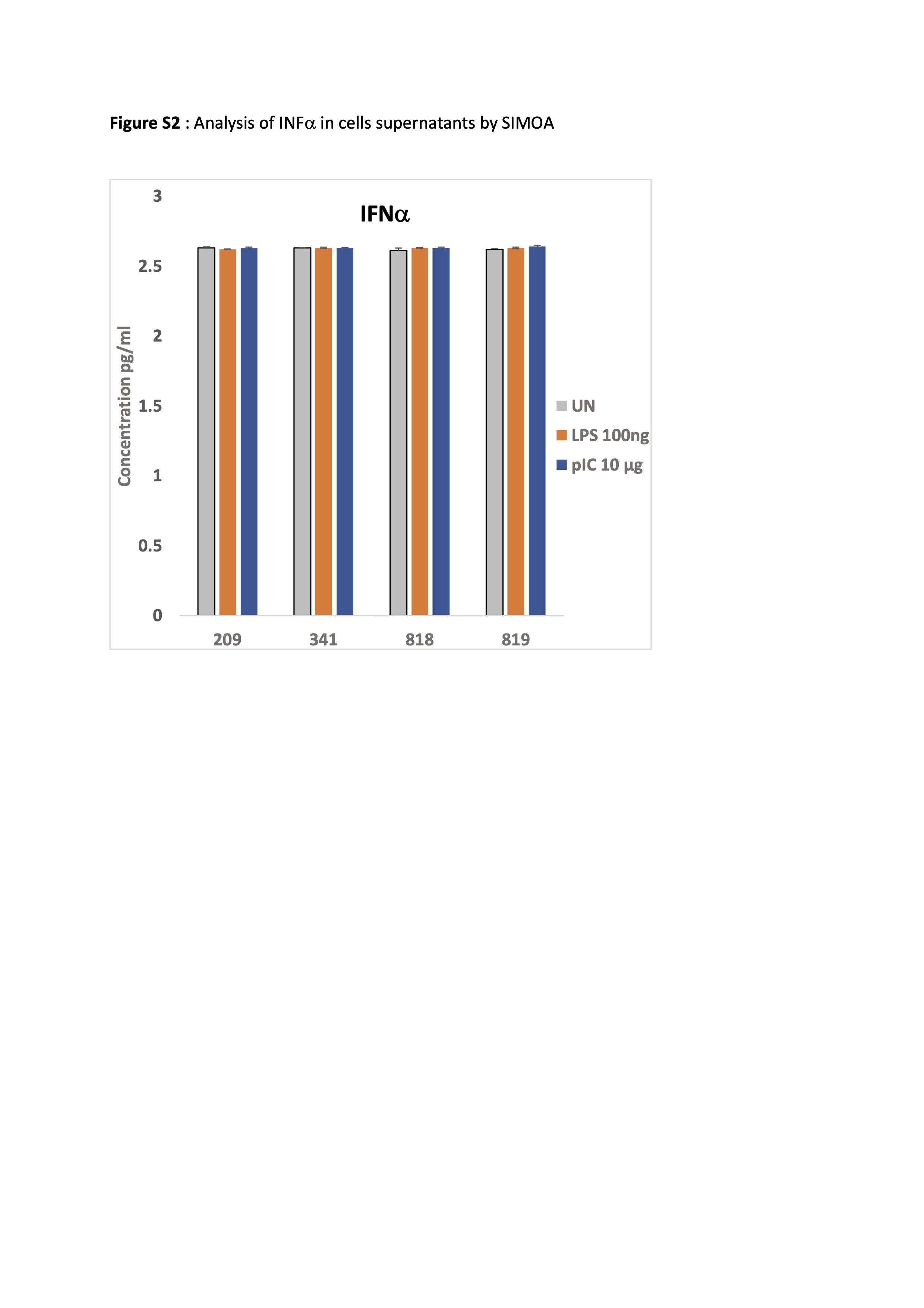

Supplement: Supplementary file 2 [file Image_2.jpeg]
